# Comparative Treatment Study on Macular Edema Secondary to Branch Retinal Vein Occlusion by Intravitreal Ranibizumab with and without Selective Retina Therapy

**DOI:** 10.3390/life13030769

**Published:** 2023-03-13

**Authors:** Manabu Yamamoto, Yoko Miura, Kumiko Hirayama, Akika Kyo, Takeya Kohno, Dirk Theisen-Kunde, Ralf Brinkmann, Shigeru Honda

**Affiliations:** 1 Department of Ophthalmology and Visual Science, Osaka Metropolitan University Graduate School of Medicine, Osaka 545-8585, Japan; 2 Institute of Biomedical Optics, University of Lübeck, 23562 Lübeck, Germany; 3 Department of Ophthalmology, University of Lübeck, 23562 Lübeck, Germany; 4 Medical Laser Center Lübeck, 23562 Lübeck, Germany

**Keywords:** retinal laser therapy, branch retinal vein occlusion, macular edema, selective retina therapy, anti-VEGF therapy, ranibizumab

## Abstract

The purpose of this study was to compare the safety and efficacy of selective retina therapy (SRT) combined with the intravitreal injection of ranibizumab (IVR) in patients with macular edema (ME) secondary to branch retinal vein occlusion (BRVO). This trial was a 12-month single-center, randomized, single-masked prospective study. Eligible patients were randomized (1:1) to IVR and SRT (IVR + SRT group), or IVR and sham SRT (IVR + sham group). After the initial IVR, all participants received ME resolution criteria-driven pro re nata treatment. SRT or sham SRT was always applied one day after IVR. The primary outcome measure of this study was the mean change in central macular thickness (CMT) from baseline, and the secondary outcome measures were the mean change in visual acuity from baseline and the number of IVR treatments at a 52-week follow-up. Thirteen patients were in the IVR + SRT group, and 11 were in the IVR + sham group. Compared to the baseline, mean CMT and BCVA improved significantly after 52 weeks in both groups, with no significant difference between the two groups. The mean number of IVR was 2.85 ± 1.52 in the IVR + SRT group and 4.73 ± 2.33 in the IVR + sham group at the 52-week follow-up, with a significant difference between the two groups (*p* < 0.05). IVR combined with SRT may significantly decrease the number of IVR treatments while maintaining the visual and anatomical improvement effect of IVR monotherapy.

## 1. Introduction

Retinal vein occlusion (RVO) is the second most common disease of retinal vascular disease after diabetic retinopathy, classified as either branch retinal vein occlusion (BRVO) or central retinal vein occlusion (CRVO) depending on the location of the occlusion [1]. The worldwide incidence of RVO is estimated to be 16.4 million people, of which about 80% are BRVO patients. Macular edema (ME) is a major complication of RVO that results in significant visual impairment [2]. There have been a number of treatment modalities advocated for the management of BRVO, including laser treatment, the intravitreal or periocular application of steroids, sheathotomy and vitrectomy, and the intravitreal injection of vascular endothelial growth factor (VEGF) inhibitors [3,4,5,6,7,8,9,10].

VEGF inhibitors, including ranibizumab and aflibercept, have been the first choice of therapeutic options for ME secondary to RVO, targeting the molecules responsible for the increased vascular permeability. A number of large clinical trials has reported that these VEGF inhibitors contribute to improved visual acuity and retinal anatomy for ME associated with RVO [8,9]. Although this varies depending on the treatment regimen, a large number of repeated anti-VEGF treatments is required to maintain improved vision. Repeated anti-VEGF intravitreal injections may be causing a wide range of burdens for patients and health care systems [11]. Therefore, seeking for ways to reduce the number of injections without diminishing their efficacy is important.

Selective retina therapy (SRT) was developed as a novel and unique laser procedure in which the RPE is selectively broken down through a microbubble formation within RPE cells [12,13,14]. This treatment minimizes thermal diffusion in surrounding tissues, which enables selective RPE disruption without damaging the neural retina or choroid. Several reports revealed that SRT was effective in improving exudative changes and maintaining visual function for macular disease, including diabetic macular edema (DME), subretinal fluid associated with central serous chorioretinopathy, tilted disc syndrome, choroidal nevus, and postoperative rhegmatogenous retinal detachment [15,16,17,18,19,20,21,22,23,24,25]. No adverse effects were reported in all these studies. These results provide clinical evidence that SRT is not only effective, but also anatomically safe, without damage to the neural retina or choroid, and also functionally safe, without compromising local retinal sensitivity. Therefore, it is very worthwhile to investigate SRT for a variety of other exudative macular diseases.

There are currently no reports of SRT treatment for ME associated with BRVO. The purpose of this study was thus to compare the safety and efficacy of the combination therapy of the intravitreal injection of ranibizumab (IVR) and SRT with IVR monotherapy in patients with ME secondary to BRVO.

## 2. Materials and Methods

### 2.1. Subjects

This trial was a 12-month single-center, randomized, single-masked prospective study designed to compare the efficacy and safety profile of SRT combination therapy with IVR and IVR monotherapy in participants with ME secondary to BRVO. It was performed in Osaka Metropolitan University Hospital (formerly Osaka City University until 31 March 2022). This study started in July 2014 and was completed in 2017. This study was approved by the Ethical Committee of Osaka Metropolitan University Graduate School of Medicine (protocol code 2844, date of approval: 30 May 2014), carried out on the basis of the Declaration of Helsinki, and SRT was registered with the University Hospital Medical Information Network (UMIN) (ID. UMIN000028347). Written informed consent was obtained from all patients prior to enrollment.

### 2.2. Inclusion and Exclusion Criteria

The inclusion criterion for SRT was ME secondary to BRVO giving rise to subjective symptoms, such as central scotoma, metamorphopsia, and reduced visual acuity. Patients with a central macular thickness (CMT) > 300 µm measured by optic coherence tomography (OCT) were included.

The ophthalmological exclusion criteria were as follows:

(1) Loss of sight in one eye;
(2) Optic media that are insufficiently transparent to acquire fundus images or obtain other imaging findings from the eye to be treated;
(3) Presence of inflammatory intraocular disorders, including infectious disorders;
(4) Intraocular surgery or laser treatment within 6 months;
(5) Intravitreal injection within 3 months;
(6) Presence of a comorbidity reducing the visual acuity of the eye to be treated or that may require medical or surgical treatment during the study period;
(7) Ophthalmic impairment in the eye to be treated that would confuse interpretation of the effectiveness of treatment in the judgment of an investigator or subinvestigator;
(8) Scarring or atrophy of the central fovea indicating that reduced visual acuity of the eye to be treated would not be recoverable;
(9) Vitreous traction or epiretinal membrane in the eye to be treated, visible on biological optical microscopy or OCT, that would significantly affect central visual acuity in the judgment of an investigator or subinvestigator;
(10) Neovascularization of the iris or vitreous hemorrhage in the eye to be treated;
(11) History of hypersensitivity to any component of anti-VEGF drugs.

The systematic exclusion criteria were as follows:

(1) Inflammatory disease;
(2) Bleeding tendency and anticoagulation therapy;
(3) Presence or possibility of pregnancy;
(4) Untreated hypertension and diabetes mellitus;
(5) History of stroke (cerebral infarction, cerebral hemorrhage, etc.) or transient ischemic attack.

### 2.3. Treatment Strategy

**Figure 1:**
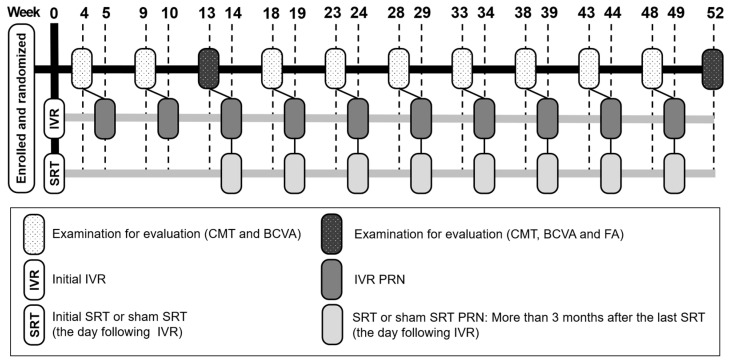
Schema of the treatment schedule for this study.

Figure 1 shows the treatment schedule of this study. All eligible patients were randomized (1:1) to either combination therapy with IVR and SRT (IVR + SRT group), or IVR and sham SRT (IVR + sham group). Randomization was performed using the random number table. One IVR was administered at baseline, followed by a monthly pro re nata (PRN) regimen. After topical anesthesia of 0.4% oxibuprocaine, intravitreal injection of 0.5 mg/0.05 mL ranibizumab was performed into the sclera 3.5 mm from the corneal limbus. SRT and sham SRT were performed the day following IVR. The retreatment criteria were confirmation of ME with a CMT of 300 μm or greater, or with a CMT increased 20% from the last evaluation. Retreatment with IVR was performed 1 week after examination day. Retreatment with SRT and sham SRT was administered on a PRN regimen basis on the IVR, with at least a 3-month interval between SRT treatments.

### 2.4. SRT Method

All SRT laser irradiations were conducted by a single ophthalmologist. The SRT system (Medical Laser Center Lübeck, Lübeck, Germany) utilizes a Q-switched neodymium-doped yttrium lithium fluoride (Nd:YLF) laser, frequency-doubled to a wavelength of 527 nm. In a single irradiation, a short 1.7 µs laser pulse is repeated 30 times at a repetition rate of 100 Hz. The laser beam was adjusted such that the irradiation diameter on the retina was approximately 200 µm, with a top-hat beam profile under the use of a 1.05× magnification Mainster central field contact lens.

Additionally, a real-time optoacoustic dosing control was used. It detects the pressure waves emitted after microbubble formation within the RPE, which are responsible for the selective RPE disruption. The pressure waves were measured with an ultrasonic transducer embedded in the Mainster contact lens (Medical Laser Center Lübeck, Lübeck, Germany) and processed using a software that calculated an optoacoustic value (OA value) after each exposure. The OA value was found to correlate with angiographic leakage. A detailed description of optoacoustic dosimetry was published by Schuele et al. [26]. The OA values for 50% and 90% probability (ED50 and ED90, respectively) for bubble detection with the used system are known through our previous studies as 70 and 112, respectively [18,19,22,23,24]. The treatment proceeded, while simultaneously, the OA value was recorded for each laser spot. In case the OA value was below ED90, the same location was irradiated again with an increased pulse energy until the ED90 OA value was first exceeded, so that RPE cell disruption with a minimally required energy was assured. Prior to SRT, the location of the macular edema was identified using FA and OCT to determine the extent of laser irradiation. The area of the edema was covered in a grid pattern, with the spacing between spots of about one spot in diameter (200 µm). Sham SRT was applied with the SRT laser, while only using the aiming beam, without the emission of the treatment laser light.

### 2.5. Clinical Observations

All participants underwent the following ophthalmic observations at baseline and monthly after the treatment: the best corrected visual acuity (BCVA) measurement, slit-lamp microscopy, funduscopy, OCT (SPECTRALIS^®^; Heidelberg Engineering GmbH, Heidelberg, Germany), color fundus photography, and fundus autofluorescence. Fluorescein angiography (FA) (SPECTRALIS^®^) was performed at screening, which was at week 13 and week 52. For the BCVA analysis, decimal visual acuities were converted to logarithmic minimum angle of resolution (logMAR) values.

### 2.6. Outcome Measures

The primary outcome measure of this study was the mean change in CMT from baseline at week 52. The secondary outcome measures included the mean change in BCVA from baseline, change in leakage on FA at week 52, and number of IVR and SRT treatments in 12 months. Adverse events (AEs) were assessed at each visit.

### 2.7. Statistical Analysis

Changes in CMT and BCVA from baseline were assessed using a Wilcoxon signed-rank test. The Mann–Whitney U test was used for comparison between the groups in CMT, BCVA, and number of IVR treatments. The chi-square test was used for comparison between the groups in leakage on FA. IBM^®^ SPSS^®^ Statistics 24.0 (IBM Japan, Ltd., Tokyo, Japan) was used for statistical analysis, in which *p* values < 0.05 were regarded as significant.

## 3. Results

**Figure 2:**
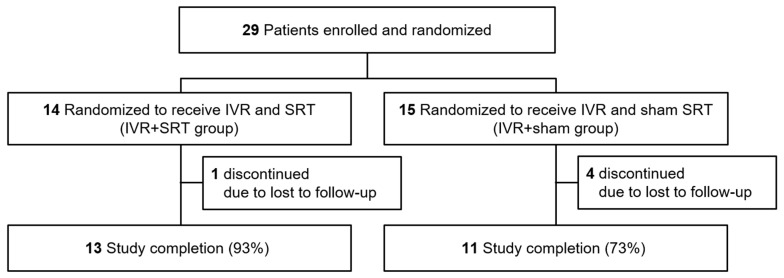
Study follow-up flow chart.

**Figure 3:**
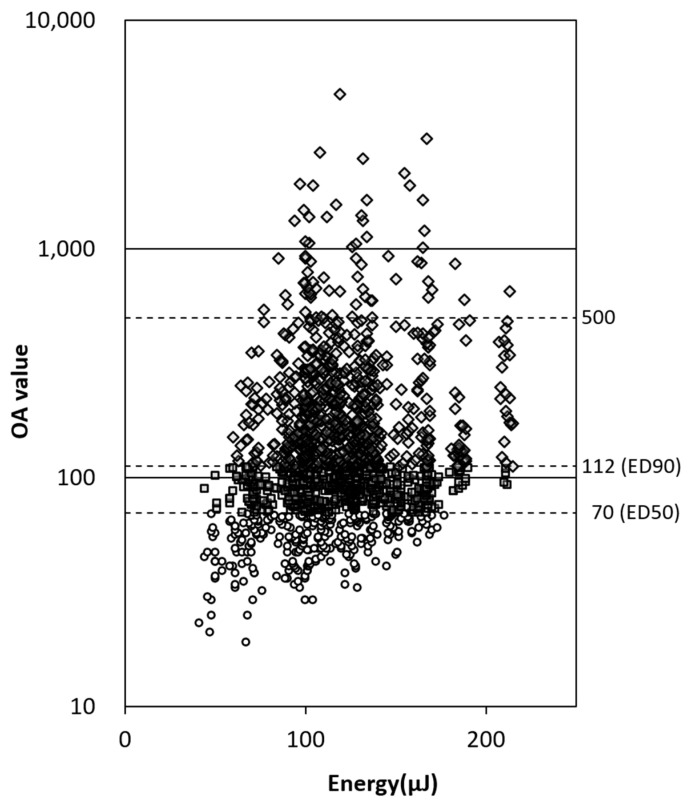
SRT irradiation energy and optoacoustic (OA) values. The scatter plot shows the correspondence between irradiation energy per pulse and optoacoustic value at each irradiation spot in all cases. Circles (274 points), squares (538 points), and diamonds (735 points) refer to OA values below ED50, ED50 to 90, and ED90 and above, respectively. The dashed lines represent the ED50 and ED90 OA values for the detection of microbubble formation.

In total, 29 participants were enrolled and randomized into the IVR + SRT group (n = 14) and IVR + sham group (n = 15). Five participants (one in the IVR + SRT group and three in the IVR + sham group) discontinued this study before week 52. Overall, 24 participants completed the 12 months of this study (13 in the IVR + SRT group and 11 in the IVR + sham group) (Figure 2). Table 1 shows the baseline characteristics of the patients in this study. Full details of all participants are provided in the supplementary data (Table S1). There were no significant differences in baseline characteristics between the two groups. The number of SRT laser spots per treatment day was 55.3 ± 17.6 (31 to 102) on average over all patients, of which 19.2 (34.8%) and 26.3 (47.5%) were above the ED50 and ED90 OA values, respectively. The number of spots for a single patient was kept approximately constant during retreatment. Since SRT spots are ophthalmoscopically invisible, irradiation of the same area as in previous treatments could not be excluded. The pulse energies used, on average over all treatments, were 117.9 ± 28.0 µJ (185.3 ± 75.8 in OA values) (Figure 3).

**Figure 4:**
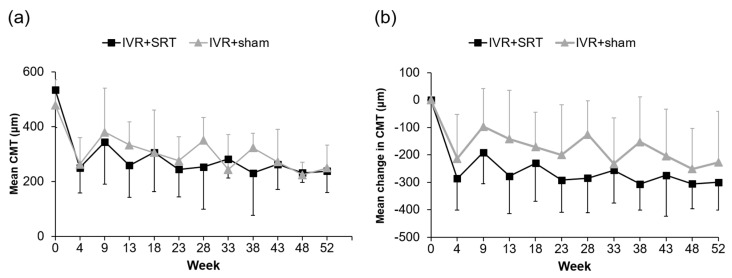
Mean CMT (**a**) and mean change in CMT (**b**) from baseline to week 52.

The mean CMT in the IVR + SRT and IVR + sham groups was 534 ± 92 µm and 479 ± 138 µm, respectively, at baseline; 259 ± 84 µm and 333 ± 117 µm, respectively, at the 13-week follow-up; and 237 ± 83 µm and 249 ± 78 µm, respectively, at the 52-week follow-up. A significant difference was seen in both groups between the means at baseline and at the 52-week follow-up (IVR + SRT: *p* < 0.01; IVR + sham: *p* < 0.01). The mean change in CMT was −297 ± 100 µm in the IVR + SRT group and −230 ± 182 µm in the IVR + sham group, with no significant difference (*p* = 0.26) (Figure 4). 

**Figure 5:**
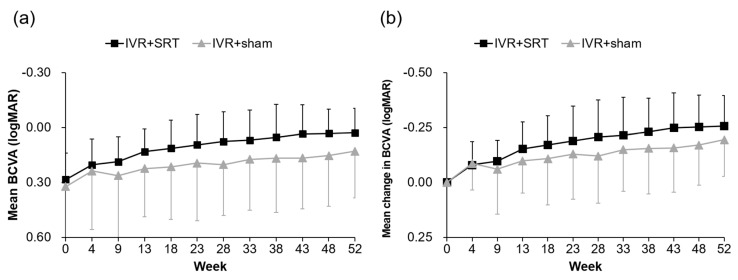
Mean BCVA (**a**) and mean change in BCVA (**b**) from baseline to week 52.

The mean BCVA in the IVR + SRT and IVR + sham groups was 0.28 ± 0.14 and 0.32 ± 0.31, respectively, at baseline; 0.13 ± 0.12 and 0.22 ± 0.26, respectively, at the 13-week follow-up; and 0.03 ± 0.13 and 0.13 ± 0.26, respectively, at the 52-week follow-up. A significant difference was seen in both groups between the means at baseline and at the 52-week follow-up (IVR + SRT: *p* < 0.01; IVR + sham: *p* < 0.01). The mean change in BCVA was −0.26 ± 0.14 in the IVR + SRT group and −0.19 ± 0.17 in the IVR + sham group, with no significant difference (*p* = 0.10) (Figure 5).

**Figure 6:**
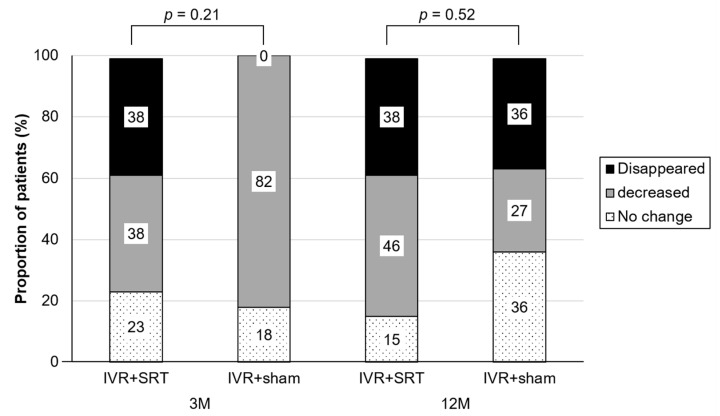
Change in macular leakage on FA.

Leakage on FA in the IVR + SRT and IVR + sham groups disappeared in 38% and 0%, decreased in 38% and 82%, and was unchanged in 23% and 18%, respectively, at the 13-week follow-up; and disappeared in 38% and 36%, decreased 46% and 27%, and was unchanged in 15% and 36%, respectively, at the 52-week follow-up, with no significant difference (13-week: *p* = 0.21; 52-week: *p* = 0.52) (Figure 6).

The mean number of intravitreal injections of ranibizumab was 1.62 ± 0.65 in the IVR + SRT group and 1.82 ± 0.75 in the IVR + sham group from week 0 to the 13-week follow-up; 1.23 ± 1.24 and 2.91 ± 1.87, respectively, from week 14 to the 52-week follow-up; and 2.85 ± 1.52 and 4.73 ± 2.33, respectively, from week 0 to the 52-week follow-up. A significant difference was seen between the two groups from week 0 to the 13-week follow-up and week 0 to the 52-week follow-up (*p* < 0.05 and *p* < 0.05, respectively). The mean number of SRT treatments was 2.15 ± 1.14 in the IVR + SRT group and 2.91 ± 0.94 in the IVR + sham group (with a sham laser) from week 0 to the 52-week follow-up (Table 2). In 2 of 154 visits (1.3%) in the IVR + SRT group and 1 of 121 visits (0.8%) in the IVR + sham group, IVR was not performed for compelling reasons despite meeting the criteria. On all three occasions, the criteria for reapplication of treatment were met at the next visit, and the patient was reapplied with IVR as originally scheduled.

During this study, no AEs were developed, such as cerebral infarction, myocardial infarction, or other systemic diseases, or intraocular inflammation, hemorrhage, or other events attributable to SRT and IVR treatment.

## 4. Discussion

This is the first report to investigate the effect of SRT combined with IVR on ME secondary to BRVO. There was no significant difference in the reduction in ME or improvement of visual acuity in the IVR + SRT group compared to the IVR + sham group. On the other hand, the IVR + SRT group had significantly fewer treatments in 52 weeks than the IVR + sham group (IVR + SRT: 2.85 times; IVR + sham: 4.73 times). These results indicate that SRT combined with anti-VEGF therapy for ME secondary to BRVO may reduce the number of anti-VEGF treatments without compromising the functional and anatomical improvement effect of anti-VEGF therapy.

Table 3 shows some of previous clinical studies in which retinal laser therapy was applied to ME caused by BRVO. Retinal laser photocoagulation is considered as a well-established method for treating ME secondary to BRVO. A lot of studies have been published on the role of laser photocoagulation in the management of ME secondary to BRVO [3,27,28,29]. Several studies have been conducted to explore the optional effects of combined laser photocoagulation on anti-VEGF treatment [30,31,32]. However, these studies of conventional laser photocoagulation combined with anti-VEGF therapy did not establish any advantage in terms of functional or anatomic improvement or the reduction in the number of anti-VEGF treatments. Recently, subthreshold micropulse laser therapy (SMT) has been shown to be safer and superior to conventional laser photocoagulation and has been inferred to be as effective as anti-VEGF therapy and may reduce the number of anti-VEGF injection when used in combinatino therapy [33,34]. A systematized review and critical approach of the SMT for ME also suggested that it is possible that SMT plus anti-VEGF therapy might require fewer intravitreal injections than anti-VEGF monotherapy, with equally good functional and morphological results [35,36]. Both of these systematic reviews jointly concluded that the current literature on SMT in RVO is limited by the paucity of high-quality prospective studies, the lack of control groups, the lack of long-term observation beyond 12 months, relatively small sample sizes, and non-standardized protocols across studies. It is obvious that the effectiveness of treatment varies depending on the types of lasers and irradiation methods, so the establishment of protocols appropriate for each treatment method is desired.

SRT selectively destroys the pigmented RPE without any thermal damage to surrounding tissues, and a rejuvenation of the RPE is expected through the RPE healing process. It is suggested that this leads to the normalization and reactivation of RPE cell functions, such as the regulation of VEGF production, pump function, and outer blood–retinal fenestration [13]. Several clinical studies of SRT for CSC and DME have shown that SRT monotherapy can reduce subretinal and intraretinal fluid and improve visual function, and these are thought to be the result of the aforementioned mechanisms on RPE cells [15,16,17,18,19,20,21,22]. In our previous study, CSC and DME also showed improvement in exudative fluid 3 to 6 months after SRT, indicating that the effect lasts longer than 1 to 2 months as in anti-VEGF therapy. In the current study, the comparison of the number of treatments separated by time period showed no difference at 0–13 weeks and was significantly less in the IVR + SRT group at 14–52 weeks. This may be considered as due to a long-lasting effect of SRT in ME secondary to BRVO, as in previous reports.

In considering how to combine anti-VEGF therapy with retinal laser treatment, such as SRT, there are two issues to be discussed: the treatment’s mechanism and its long-term efficacy. As mentioned previously, this combination of therapies may have a synergistic effect on exudative changes in retinal disease, and this is attributed to differences in the mechanisms by which exudative lesions are improved. The main effect of anti-VEGF therapy is to regulate an excess permeability of the retinal vessels and reduce leakage into the neuronal retina [37]. The half-life of ranibizumab in the vitreous body is 2.88 days [38], and the therapeutic effect demonstrated in clinical trials lasts 1–2 months per treatment. On the other hand, SRT initially induces an inflammatory response associated with necrosis of the RPE, triggering RPE rejuvenation, followed a week later by the suppression of inflammatory mediators, such as complement components [39]. Since anti-VEGF has been shown to reduce the inflammatory response in macular edema [40], it would be useful to perform SRT within a few days after the intravitreal injection of anti-VEGF agents, if the suppression of post-SRT inflammatory reactions is desired. However, due to the lack of exploration into the interaction between anti-VEGF therapy and SRT in RPE cells, it is currently impossible to determine whether this suggested treatment timing is optimal. In terms of treatment stability, the number of SRT irradiations per area and their size are controversial. In the current study, SRT was performed in a grid pattern within the retinal edematous lesion, with the spacing between spots of about one spot in diameter, which was 200 µm. As shown by the relationship between irradiation energy and OA values in Figure 3, OA values ranged at the same energy level. This difference may be attributed to the local variations of clarity of the cornea, lens, and vitreous in each individual case, as well as the pigment concentration of the RPE and the degree of macular edema at the irradiation area. Although not noted in the results, many patients had residual edema, especially with regard to retinal edema, the day after the intravitreal injection of anti-VEGF agents. Basic and clinical studies are needed to determine the optimal treatment method, considering these mechanisms and stability.

Interestingly, in the current study, leakage on FA did not differ between the two groups at the 13-week and 52-week periods. Macular edema secondary to BRVO is caused by the release of substances that increase vascular permeability, such as VEGF, into the retina due to the deterioration of tight junctions between capillary endothelial cells and due to adhesions between the vitreous and retina, which damage the blood–retinal barrier [2]. VEGF inhibitors prevent increased permeability of retinal vessels by suppressing VEGF and subsequent leakage of effusion into the neuroretina [38]. FA is useful in assessing the permeability of the blood–retinal barrier, which is an important morphologic measure of ME treatment. Roider et al. performed SRT for DME and reported that functional and anatomic improvement or stabilization was achieved in 84% of patients [21]. However, there was no correlation between visual acuity and leakage on FA after SRT, which they hypothesized was because FA does not image the important fluid drainage to the choroid [21]. This may be the reason why our results also showed no difference in evaluation on FA between the two groups.

## 5. Conclusions

In conclusion, the results of the current study revealed that SRT combined with IVR significantly decreased the number of IVR treatments while maintaining the visual and anatomical improvement effect of IVR monotherapy. This study was limited by the inclusion of a small number of patients and its single-blind design. Further prospective studies with a larger number of patients and long-time follow-up are warranted to confirm the therapeutic efficacy of SRT for ME secondary to BRVO.

## Figures and Tables

**Table 1:** Patient characteristics at baseline.

Characteristics	IVR + SRT	IVR + sham	*p*-Value
Number	13 eyes	11 eyes	
Sex, male: female (cases)	7: 6	4: 7	0.33
Age; mean (range)	67 (50–85)	72 (63–80)	0.20
Hypertension (%)	9 (69)	9 (82)	0.41
Diabetes mellitus (%)	3 (23)	1 (9)	0.36
Hyperlipidemia (%)	2 (15)	3 (27)	0.41
Smoking (%)	3 (23)	6 (55)	0.12
Intraocular lens (%)	1 (8)	2 (18)	0.43
Previous treatment (%)	6 (46)	5 (45)	0.50
BCVA (logMAR); mean (SD)	0.28 (0.14)	0.32 (0.31)	0.93
CMT; mean (SD)	536 (93)	479 (138)	0.24

Abbreviations: IVR = intravitreal ranibizumab, SRT = selective retina therapy, BCVA = best corrected visual acuity, and CMT = central macular thickness.

**Table 2:** Number of intravitreal injections of ranibizumab and SRT treatments.

Number of Treatments	IVR + SRT	IVR + sham	*p*-Value
IVR			
Week 0–13; Mean (SD)	1.62 (0.65)	1.82 (0.75)	0.50
Week 14–52; Mean (SD)	1.23 (1.24)	2.91 (1.87)	<0.05
Week 0–52; Mean (SD)	2.85 (1.52)	4.73 (2.33)	<0.05
SRT (or sham SRT)			
Week 0–52; Mean (SD)	2.15 (1.14)	2.91 (0.94)	-

Abbreviations: IVR = intravitreal ranibizumab, and SRT = selective retina therapy.

**Table 3:** Clinical studies on macular edema secondary to branch retinal vein occlusion using retinal laser therapy.

Author /Year	Study Design	Follow-Up	Laser Devise/Method	Findings
BVOS group, 2018 [3]	Prospective, randomized; treated or untreated control group. VA: 20/40 or worse.	3 years	Argon laser PC	Significant VA improvement in treated group. VA: 20/70 vs. 20/40–20/50. Number of lines gained: 0.23 vs. 1.33.
Tadayoni R et al., 2017 [30]	Prospective, randomized; IVR alone, IVR + PC, PC alone groups. VA: 19 to 73 letters.	2 years	Conventional PC (No laser details.)	No significant difference between IVR and IVR + PC groups. VA change: 15.0 vs. 15.4 letters. Number of injections: 11.4 vs. 11.3.
Song S et al., 2020 [31]	Prospective, randomized; IVR or IVR + PC group. VA: 24 to 73 letters.	1 year	Grid laser photocoagulation (No laser details.)	No significant difference between IVR and IVR + PC groups. VA change: 17.9 vs. 18.1 letters. Number of injections: 4 vs. 6.
Murata T et al., 2021 [32]	Prospective, randomized; IVR or IVR + PC group. VA: 19 to 73 letters.	1 year	Focal or grid short-pulse laser (Based on the ETDRS guidelines)	No significant difference between IVR and IVR + PC groups. VA change: 17.9 vs. 18.1 letters. Number of injections: 4 vs. 6.
Buyru Ozkurt Y et al., 2018 [33]	Retrospective, non-randomized; IVR or SML group. VA: 1 to 0.22 (logMAR)	1 year	Subthreshold micropulse laser (577 nm)	No significant difference between IVR and SML groups. VA: 0.57 to 0.34 vs. 0.50 to 0.33.
Terashima H et al., 2019 [34]	Retrospective, non-randomized; IVR or IVR + SML group. VA: 20/400 to 20/25	6 months	Subthreshold micropulse laser (577 nm)	Significantly less number of injections for IVR + SML group. Number of injections: 2.3 ± 0.9 vs. 1.9 ± 0.8. No significant VA improvement between IVR and IVR + SML groups. VA: 0.11 ± 0.15 vs. 0.23 ± 0.38.
The current study	Prospective, randomized; IVR or IVR + SRT group. VA: 0.7 to −0.08 (logMAR)	1 year	SRT (527 nm, 1.7 µs, 100 Hz)	

Abbreviations: VA = visual acuity, IVR = intravitreal ranibizumab, PC = photocoagulation, and SML = subthreshold micropulse laser.

## Data Availability

The data presented in this study are available in supplementary material.

## Supplementary Materials

The following supporting information can be downloaded at: https://www.mdpi.com/article/10.3390/life13030769/s1, Table S1: BRVO list.
